# The Importance of Protesters’ Morals: Moral Obligation as a Key Variable to Understand Collective Action

**DOI:** 10.3389/fpsyg.2018.00418

**Published:** 2018-03-27

**Authors:** José-Manuel Sabucedo, Marcos Dono, Mónica Alzate, Gloria Seoane

**Affiliations:** Departamento de Psicoloxía Social, Básica e Metodoloxía, Facultade de Psicoloxía, Universidade de Santiago de Compostela, Santiago de Compostela, Spain

**Keywords:** activism, collective action, morals, moral obligation, moral conviction, moral norm, political participation, protest

## Abstract

Collective action and protest have become a normalized political behavior that in many cases defines the political agenda. The reasons why people take to the streets constitute a central subject within the study of social psychology. In the literature, three precedents of protest that have been established as central to the study of this phenomenon are: injustice, efficacy, and identity. But political action is also deeply related to moral values. This explains why in recent years some moral constructs have also been pointed out as predictors of collective action. Moral variables have been introduced into the literature with little consideration to how they relate to each other. Thus, work in this direction is needed. The general aim of this research is to differentiate moral obligation from moral norms and moral conviction, as well as to compare their ability to predict collective action. In order to do so, the research objectives are: (a) conceptualize and operationalize moral obligation (Study 1, *N* = 171); (b) test its predictive power for intention to participate in protests (Study 2, *N* = 622); and (c) test moral obligation in a real context (Study 3, *N* = 407). Results are encouraging, showing not only that moral obligation is different to moral conviction and moral norm, but also that it is a more effective predictor working both for intention and real participation. This work therefore presents moral obligation as a key precedent of protest participation, prompting its future use as a variable that can enhance existing predictive models of collective action. Results regarding other variables are also discussed.

## Introduction

*Occupy Wall Street*, *Occupy London*, *Geraçao à Rasca, 15-M indignados*… Although protest was established long ago as one of the most prevalent forms of political participation, the number of street demonstrations during the last decade has risen remarkably (Kriesi, 2016; Shuman et al., 2016). The 2008 financial crisis had great impact on collective action tendencies (Likki, 2014; Klandermans and van Stekelenburg, 2016; Kriesi, 2016; Sabucedo et al., 2017a) many of them being rooted in anti-system claims (Hughes, 2011; Páez et al., 2013). Despite this increase in protests numbers being closely related to anti-system and anti-austerity claims, the normalization of protest extends to other subjects as well: the Arab Spring, anti-xenophobia marches in South Africa in 2013, Hong Kong’s Umbrella Revolution in 2014, or the recently held Women’s March in Washington at the beginning of 2018.

As a central matter in the field, collective action has been studied for a long time, which resulted in the identification and establishment of a group of variables that proved to be useful in the prediction of protest participation. Among the variables that stand out as collective action predictors we find: injustice, efficacy, and identity. These variables were first proposed by Gamson (1992) in his work about collective action frames and have been developed and integrated into solid predictive models (for further information, see Sturmer and Simon, 2004; van Zomeren et al., 2004, 2008; Tausch et al., 2011; van Stekelenburg and Klandermans, 2013). Successive studies on collective action frames derived into what is in all likelihood the most widely used predictive model of collective action, the SIMCA, that efficiently integrates the variables of identity, injustice, and efficacy (van Zomeren et al., 2008).

However, politics is also a moral topic which means that axiological principles could also influence collective action. This is why in recent years, some engaging approaches that point toward the importance of people’s morals as a motivator for participation have been proposed. That is the case of moral conviction (van Zomeren et al., 2012; Skitka et al., 2015) and moral obligation (Vilas and Sabucedo, 2012; Vilas et al., 2016). Also, besides this linkage with politics, morals had already proven to motivate a diverse range of human behavior. The most prominent case is that of moral norm that appears associated to the prediction of a wide range of behavior (Conner et al., 2003; McMillan and Conner, 2003; Moan and Rise, 2006). In this sense, and taking into account the importance that morals acquired in the field of politics, it seems adequate to wonder if a concept as widely used in the psychological literature as moral norm is also useful to understand collective action, or at least, to explore what relationship it has with the aforementioned moral concepts.

The existence of all these concepts that are – moral obligation (Vilas and Sabucedo, 2012) and moral conviction (van Zomeren et al., 2012; Skitka et al., 2015) – or may be – moral norm (Schwartz, 1973) – related to collective action compels us to explain this relationship further. Therefore, since these three concepts seem to overlap, differentiating them will clarify how relevant they are as predictors of collective action. To do so, we will first define each of the three concepts.

Considering that the literature on moral conviction (Skitka et al., 2015) points toward a certain similarity with the concept of moral norm and that both these constructs have received more attention in the literature, they will be discussed jointly and briefly. On the other hand, since moral obligation has a shorter trajectory in the field and constitutes the central proposal of this work, we will devote more attention to it. Finally, once the three concepts are defined, we will discuss their conceptual differences.

### Moral Norm and Moral Conviction

The origins of moral norm can be tracked to the work of Schwartz (1973), where he proposed the concept of personal norm (from now on referred to as moral norm, for the sake of better comprehension). The conceptualization of Schwartz’s moral norm draws from Fishbein’s (1967) work on attitude and the prediction of behavior, specifically from the personal normative belief concept, defined as “a belief of whether the particular act should or should not be performed” (Fishbein, 1967, p. 489). Moral norm was later introduced in the theory of planned behavior as an individual’s perception of a specific behavior as morally correct or incorrect (Ajzen, 1991).

In what refers to moral conviction, it is a term originally proposed by Skitka et al. (2005). It is defined as a “meta-cognitive belief that people may have about a given attitude, that is, that the attitude is grounded in core beliefs about right and wrong” (Skitka et al., 2015, p. 41). Despite the similarity with the definition of moral norm, the fact that moral convictions stand in a core position among personal beliefs makes for a qualitative difference between them and that may be enough to differentiate the two.

Additionally, as already noted, moral conviction does appear as a useful predictive variable in the literature of collective action (Skitka and Wisneski, 2011; van Zomeren et al., 2012).

### Moral Obligation

Previously, Vilas and Sabucedo (2012) referred to moral obligation as “a personal decision to participate in a specific collective action based on the belief that this is what should be done” (Vilas and Sabucedo, 2012, p. 371). Revisiting the concept, it seems more adequate to define it as a motivational force toward a certain action that later could end in a decision to execute a behavior.

The theoretical basis of moral obligation came from Kant’s work on the categorical imperative, an objective principle that must always be followed no matter the consequences (Johnson and Cureton, 2017). In the same vein, Bandura (1986) mentions that on occasion, people behave based on their moral despite the elevated costs that these actions could involve. Therefore, a sense of obligation is one of the aspects to be considered when we refer to moral obligation.

Another characteristic of moral obligation is that it constitutes a strictly personal motivation, as it is linked to personal codes of conduct. In other words, the need to comply is toward oneself and no other normative sources like the reference group. In this sense, moral obligation is endowed with a sense of autonomy. From the moment people execute these actions freely, and not forced by contextual demands, their self-concept (Higgins, 1989), well-being (Diener and Diener, 1995), and pride (Tracy and Robins, 2004) are boosted. In other words, their personal satisfaction increases.

Along with these three dimensions – sense of obligation, autonomy, and personal satisfaction – previously posited in the work by Vilas and Sabucedo (2012), there are two additional aspects that are relevant to characterize moral obligation. One of them refers to the cost of not behaving according to one’s beliefs. The theory of self-discrepancy (Higgins, 1987) posits, following the classical concept of cognitive dissonance (Festinger, 1957) that discrepancy between beliefs and actions produces psychological distress. More specifically, Higgins (1987) proposes that discrepancies between one’s actual and ought self produced guilt.

The other dimension that should be added to the conceptualization of moral obligation is that of sacrifice. This is to say, people who act guided by moral obligation may deal with personal sacrifice, one aspect that was already present in Kant’s work (Johnson and Cureton, 2017). Writing about heroism, Zimbardo (2007) states that duty, a concept similar to that of the moral obligation, takes priority over high personal costs. Costs depend on the socio-political context, but whatever they are always implies a barrier to participate in political action. Hence, our conceptualization of moral obligation comprehends these five aspects: sense of obligation, autonomy, personal satisfaction, discomfort, and sacrifice.

Accordingly, and following the arguments previously exposed, moral obligation can be understood as a personal motivation to behave according to a series of moral self-expectations of one’s conduct, which at the same time are developed over a set of personal values and ideas.

### Conceptual Differences Between Moral Norm, Moral Conviction, and Moral Obligation

The definitions of moral obligation, moral conviction, and moral norm have been discussed, and it can be observed that they share some characteristics, as, for instance, personal commitment, whether related to beliefs (moral norm and moral conviction) or to actions (moral obligation). However, differences between them also exist, and this is of capital importance since it is what gives sense to discussing their relation and their potentially different roles in the explanation of collective action.

Regarding the differences between moral norm and moral obligation, the definition of norm and obligation by The Oxford Dictionary of Philosophy may be a convenient starting point to this discussion. Norm is described as: “a rule for behavior, or a definite pattern of behavior, departure from which renders a person liable to some kind of censure […]”; while obligation is defined as: “an action that is required of one” (Blackburn, 2008, p. 267).

Taking the above mentioned into account, it could be concluded that: (a) moral norm is what defines which behavior is right and which is wrong and (b) moral obligation is the motivation felt to comply with that moral norm. In other words, and using self-discrepancy theory (Higgins, 1987) as a framework, the moral norms will be the individual’s self-guides while moral obligation will be the motivation felt to behave accordingly.

Concerning the relationship of moral norm and moral conviction, the main difference between these two constructs lies in the centrality of the attitude in question. Moral conviction is generally regarded as a particularly strong attitude based on moral content, which is perceived as nuclear to one’s beliefs (Skitka and Morgan, 2014). According to this, a moral conviction can be understood as an especially strong and important moral norm.

Regarding the difference between moral conviction and moral obligation, it is important to keep in mind that moral conviction is a special case of moral norm. Therefore, moral convictions allude to an individual’s self-guides – in the same way as moral norms do, and moral obligation is the motivation to act according to them. In this case, given that moral conviction is more central than moral norm, moral obligation must be greater when associated to the former.

The arguments expressed above point toward these three concepts clearly constituting different realities. Nevertheless, this argument must be supported by empirical data.

### General Aims and Hypotheses

The objectives of the present work are as follows: (1) to define and operationalize the concept of moral obligation, and to empirically test the proposed theoretical differences between moral obligation, moral conviction, and moral norm, as well as to analyze the relationship between those moral variables and their value as predictive variables of political participation intention (Study 1); (2) to test the role of moral obligation as a predictor of intention to participate in collective action along with the most used predictive variables in the literature, those of the SIMCA (van Zomeren et al., 2008; Study 2); and (3) to test moral obligation’s predictive power in the context of real participation in a demonstration.

The initial hypotheses are as follows: (a) moral obligation, moral norm, and moral conviction are three different realities; (b) the potential predictive impact of moral norm and moral conviction over the intention to participate in collective action will be mediated by moral obligation; (c) moral obligation constitutes the most effective predictor of collective action participation of the three; and (d) the predictive role of moral obligation will remain to be key even with the presence of variables that have already proven to be efficient predictors of collective action.

## Study 1

### Introduction

The validation of a new measure of collective action following the conceptual changes suggested is the first objective of the present research. This measure will then be compared to others of moral conviction and moral norm to test if they are different constructs. Finally, the three of them will be used to predict intention to participate in collective action in order to test which one is the best predictive variable.

### Method

#### Participants and Procedure

One hundred and seventy-one (171) Spanish participants (150 women; mean age = 20.55) voluntarily completed a questionnaire where they were asked about their political opinion on the basis of a hypothetical raise in tuition fees by the Spanish government. This procedure was adapted from van Zomeren et al. (2012). The context was given by a report elaborated for the study, which stated that a raise in tuition fees was being considered by the government. This bogus news item was included at the beginning of the questionnaire. After the participants had completed the questionnaire, they were debriefed and told the news had been fabricated for the study.

#### Measures

##### Moral norm

A moral norm scale was elaborated based on measures that assessed the moral norm through the evaluation of participant’s perception of the rightness or wrongness of behavior (Conner et al., 2003; McMillan and Conner, 2003; Moan and Rise, 2006). Respondents indicated on a seven-point scale (totally disagree to totally agree) their level of agreement regarding the rightness of acting against the portrayed measures. The final measure contained three items (Cronbach’s alpha = 0.73; see Appendix I for items).

##### Moral conviction

Moral conviction was measured with a scale from van Zomeren et al. (2012). The scale was composed of four (Cronbach’s alpha = 0.83) items and the scoring logic was the same as for the moral norm scale (see Appendix I).

##### Intention to participate

A seven-item scale was developed based on the work of (J. M. Sabucedo and Arce, 1991; Cronbach’s alpha = 0.80). Participants were asked on their intention to participate through different courses of action. Four of the items tapped conventional participation (Cronbach’s alpha = 0.71), while the other three tapped unconventional participation (Cronbach’s alpha = 0.80). Again, the score system was a seven-point Likert scale (1 “totally disagree” to 7 “totally agree”).

### Results

#### Developing the Moral Obligation Scale

Based on the recommendations of the European Federation of Psychologists’ Associations on test quality indicators (Evers et al., 2012), the scale construction process began by defining its dimensions from a theoretical approach, where it was intended to get a more exhaustive moral obligation measure than previous ones.

This process was done using the Moral Obligation Scale by Vilas and Sabucedo (2012) as the main reference. Based on this work, the dimensions of sense of obligation, autonomy, and personal satisfaction are included. As previously mentioned, two more dimensions were added to these three. The discomfort caused by not acting in accordance with one’s own moral (Stets and Carter, 2012), and sacrifice (Johnson and Cureton, 2017) are the dimensions that complete the conceptualization of the Moral Obligation Scale.

A group of three experts was gathered and each of them was asked to independently develop a total of five items per dimension. The resulting items were evaluated and selected upon agreement among the same experts. This resulted in a total of 16 items (*M* = 4.47; SD = 1.03; Cronbach’s alpha = 0.92) that were later submitted to statistical filtering.

#### Reducing the Moral Obligation Scale

An important concern regarding the elaboration of the Moral Obligation Scale was its fit to be used in the context of a real demonstration. This circumstance requires brief measure instruments; therefore, obtaining a short scale was a capital goal of this study.

An exploratory factor analysis (EFA) with varimax rotation was performed on the original 16-item scale. Results of this analysis showed four factors, close to the original concept that proposed five dimensions of moral obligation. Two items were selected from the first factor, which grouped two of the original dimensions – sense of obligation and autonomy – and one item was selected from each of the other three factors. This selection was done based on the theoretical adequacy of the items and their statistical significance, while also trying to preserve each of the five theoretically proposed dimensions. Item 1 of the final scale was selected for the dimension of sense of obligation, item 2 represented personal satisfaction and item 3 accounted for discomfort, item 4 was selected to feature in the sacrifice dimension, and finally item 5 taps for autonomy. An additional EFA on this scale showed only one factor, which supports the idea that the items account for the same concept of moral obligation.

Additionally, a confirmatory factor analysis (CFA) performed with the SPSS AMOS tool was applied on the resulting scale. Results showed a good fit of the model, beginning with a non-significant Chi-square statistic [χ^2^(5) = 4.21, *p* = 0.519]. Other indicators also accounted for a very good fit of the data: GFI = 0.99, CFI = 1.00, RMSEA = 0.00, and SRMR = 0.02. Finally, the reliability of this Moral Obligation Scale was tested, obtaining a good indicator (Cronbach’s alpha = 0.80; see the final version of the scale on Appendix I) (see Supplementary Data Sheet S1 for the original data matrix).

#### Empirical Differences Between: Moral Norm, Moral Conviction, and Moral Obligation

As previously stated, the concepts of moral obligation, moral norm, and moral conviction were supposed to be three different realities. However, it was also theorized that they were also closely related. This relationship is to be expected due to the nature of the constructs and does not contradict the fact that the three of them could be different constructs. The data showed such closeness, correlation of moral obligation and moral conviction was fairly high (*r* = 0.627, *p* < 0.001), while the correlation between moral obligation and moral norm was lower, though also significant (*r* = 0.414, *p* < 0.001).

Despite this, theoretical differences were proposed that were ought to be empirically tested. To do so, an EFA was performed over the items of the three constructs. The results of this analysis showed three clearly different factors, which comprised each of the items of the original scales (see **Table 1**).

**Table 1:** Factor loadings for the moral scale items.

	Component		
	
	1	2	3
Moral Obligation_1	0.752	0.215	0.194
Moral Obligation_5	0.729	0.374	0.216
Moral Obligation_3	0.682	0.272	0.180
Moral Obligation_2	0.669	–	0.162
Moral Obligation_4	0.608	0.363	–
Moral Conviction_2	0.246	0.871	–
Moral Conviction_1	0.121	0.867	0.150
Moral Conviction_3	0.281	0.702	0.188
Moral Conviction_4	0.427	0.625	–
Moral Norm_2	0.109	–	0.908
Moral Norm_1	0.255	–	0.860
Moral Norm_3	0.141	–	0.640


*Factor loadings under 0.10 are not displayed.*

**FIGURE 1:**
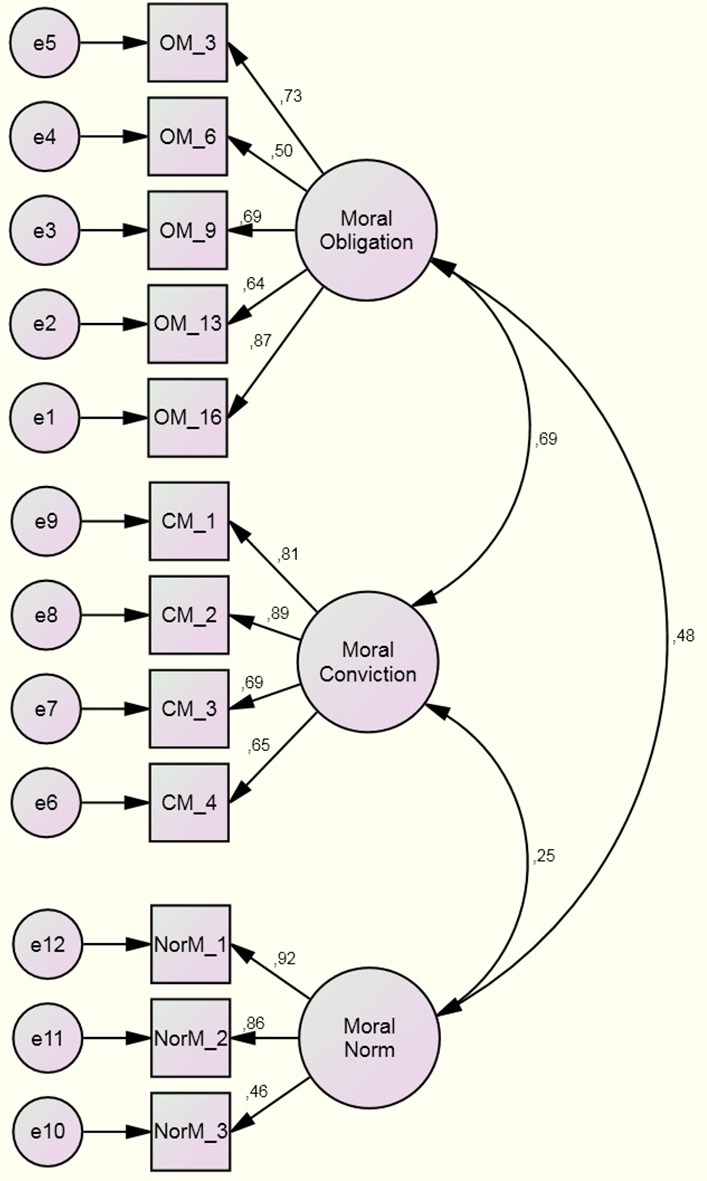
Confirmatory factor analysis results for Study 1.

A CFA was also executed to test the hypothesized model formed by the three differentiated constructs of moral obligation, moral conviction, and moral norm (see **Figure 1**). Results of the analysis showed a good fit although the Chi-squared test was statistically significant [χ^2^(51) = 80.70, *p* = 0.005]. The fact that Chi-square was significant requires the use of other indicators to assess the fit of the model; those indicators showed a good fit of the model: GFI = 0.92, CFI = 0.96, RMSEA = 0.059, and SRMR = 0.05. These data indicate that moral obligation, moral norm, and moral conviction are indeed distinct. An alternative model was tested, where all the variables from the three constructs loaded into a single construct. This was done as it was understood to be the converse scenario to the one formerly tested. The fit of that model was poor, suggesting that all the items did not form a single construct: GFI = 0.74; CFI = 0.67; RMSEA = 0.17; and SRMR = 0.11.

#### Predicting Intention to Participate Through Moral Obligation

To test the predictive power of the Moral Obligation Scale on collective action tendencies, a step-wise linear regression analysis was conducted on the intention to participate using moral obligation, moral conviction, and moral norm as independent variables.

Results showed a first significant model where moral obligation was the only predictor of intention to participate [*F*(1,169) = 89.52, *p* < 0.001] where *R*^2^ was 0.34. The next step included moral conviction [*F*(2,168) = 48.08, *p* < 0.001] where *R*^2^ was equal to 0.35. In the case of moral norm, this variable did not enter the step-wise analysis (see **Table 2**).

**Table 2:** Linear regression analysis results (Study 1).

Model		*B*	Std. error	β	*t*	Sig.
1	Moral obligation	0.498	0.053	0.588	9.46	<0.001
2	Moral obligation	0.407	0.067	0.481	6.09	<0.001
	Moral conviction	0.148	0.067	0.171	2.16	0.032


*Dependent Variable: Iintention to participate. Moral norm did not enter in the step-wise linear regression analysis.*

Two additional analyses were performed, with conventional and non-conventional participation as the dependent variables. When the dependent variable was changed to conventional participation intention, the step-wise regression resulted in a final three variable model [*F*(3,167) = 51.69, *p* < 0.001] comprising moral obligation, moral conviction, and moral norm (from higher to lower β), while *R*^2^ was 0.47 Meanwhile, when the dependent variable was non-conventional participation intention, only moral obligation accounted for it [F(1,169) = 36.39, *p* < 0.001], with the rest of dependent variables left out of the analysis; here, *R*^2^ was 0.17.

Aside from the previous data, it is interesting to analyze the relationship between moral norm, moral conviction, moral obligation, and collective action intention. Thus, a mediation analysis was performed in order to explore if moral norm and moral conviction precede of moral obligation. This analysis was carried out with the SPSS PROCESS macro [version 3.0; for additional information on the software, see (Hayes and Preacher, 2014)]. A first model was performed to test if moral obligation mediated the effect of moral norm on intention to participate. The indirect effect was tested through bootstrap estimation (1000 samples, 95% level of confidence). The effect of the mediation of moral obligation was significant, as 65% of the effect moral norm had on intention to participate was due to the mediational route of moral obligation (*P*_M_ = 0.65, SE = 0.19, 95% CI = 0.38, 1.10), whereas the inverse model of moral norm mediating the effect of moral obligation produced a non-significant indirect effect; in other words, the mediation of moral norm had no impact in how moral obligation explained intention to participate (95% CI = 0.00, 0.22).

Another mediation tested the effects of moral obligation as a mediator in the relationship between moral conviction and intention to participate. The percentage explained by the mediational path over the total effect was, in this case, a 63% (*P*_M_ = 0.63, SE = 0.14, 95% CI = 0.39, 0.98). An alternative model was performed where moral conviction mediated the effect of moral obligation on intention to participate. Here the mediation of moral conviction was responsible for 18% of the total effect explained in the model (*P*_M_ = 0.18, SE = 0.10, 95% CI = > 0.00, 0.42). This indicates that the role as a mediator of moral obligation on moral conviction is greater than that of moral conviction on moral obligation.

Finally, the same procedure was applied to the relation between moral norm and moral conviction. When moral conviction mediated the effect of moral norm, the mediational route accounted for 30% of the total effect (*P*_M_ = 0.30, SE = 0.12, 95% CI = 0.12, 0.63) while when moral norm mediated moral conviction that percentage was down to 12% (*P*_M_ = 0.12, SE = 0.05, 95% CI = 0.04, 0.40).

### Discussion

Results of Study 1 support the initial hypothesis that presented moral obligation, moral conviction, and moral norm as differentiated constructs. This could mean a great deal, not only in the field of collective action but also in other areas of study within the body of social psychology. The theory of planned behavior (Ajzen, 1991) is a good example; here moral norm is usually operationalized as perceived moral obligation, while our results point toward the idea that these two concepts are different even though closely related, which may require reconsidering the adequacy of measuring moral norm with items of moral obligation.

Also, a reliable, short scale to measure moral obligation was obtained. This scale could easily be used in field studies of real demonstrations, where briefness of measurement instruments is capital.

The data of Study 1 also support the idea that among the three different concepts that account for moral values, moral obligation is the one that works as the best predictor. This might be because it is conceptually closer to the actual manifestation of behavior than the other two. While the moral norm is conceived as the conception of a determined behavior as morally right or wrong, and moral conviction represents what could be a particularly central moral norm among the person’s belief system, the concept of moral obligation accounts for the motivation felt by the individual to behave accordingly to those moral norms.

The theoretical argument of moral norm and moral conviction being precedents of moral obligation seems to be supported by the evidence of the mediational analyses. The fact that moral obligation plays a bigger role as a mediator of moral conviction and moral norm than vice versa is what gives support to this argument. Also, moral conviction being a better mediator of moral norm than moral norm is of moral conviction also gives some support to the idea of moral conviction being a special kind of moral norm. Despite this, it has to be noted that causal assumptions derived from mediational analysis should be addressed with caution (Spencer et al., 2005).

The fact that the sample should be more heterogeneous in terms of age, as well as more balanced in terms of sex constitutes a limitation of the present study, but nonetheless, it is considered as adequate considering the objectives set in its inception.

These results also encourage the use of the Moral Obligation Scale along with other predictors of collective action, to see how it fares when integrated among other, non-moral nature predictive variables of collective action.

## Study 2

### Introduction

Following the discussion of Study 1, the main objective of Study 2 is to test how the moral obligation variable fares when predicting participation intention while acting together with other predictive variables. The intention was also to perform this analysis with an ampler and more heterogeneous sample, to cover for the limitations of the previous study.

The variables that will feature in Study 2 are those derived from the SIMCA: injustice, efficacy, and identity. All of them should have a predictive impact on the intention to participate, following the findings of van Zomeren et al. (2008). Additionally, moral conviction will also feature in this study, but not moral norm. The reason for this is that in Study 1 moral norm did not predict neither general intention to participate nor non-conventional intention to participate – it is only very marginally predicted conventional intention to participate – when introduced along moral conviction and moral obligation in the regression analyses. Thus, moral conviction is considered the only variable of the two that could possibly work better than moral obligation as a predictor of intention to participate in collective action.

### Method

#### Participants and Procedure

A professional, specialized company was contacted to carry out the sampling for this study through an online survey directed to the Spanish general population. A sample of 625 participants was determined. That number was fixed for an infinite population, with a confidence coefficient of 95% and a sampling error of 4%. The final sample, after controlling and filtering for missing values, was of six hundred and twenty-two (622) participants from Spain’s general population, of whom 396 were women (accounting for 63.7% of the sample). Age of the participants ranged from 19 to 75, with a mean value of 43.79, which is extremely close to Spain’s actual mean age of 43.69 (Instituto Nacional de Estadística, 2017). Participants responded to an online questionnaire developed in Qualtrics software.

To frame the hypothetical protest, a text about the situation of Spain’s public health system appeared before the questionnaire. It was a small compendium of some of the cuts made by the government over recent years.

#### Measures

##### Moral obligation

Moral obligation was measured using the previously validated scale in Study 1. This five-item Likert scale (ranging from 1 *totally disagree* to 5 *totally agree*) showed higher levels of reliability than in the previous study (Cronbach’s alpha = 0.92).

##### Identity

Social identity was measured by tapping in the dimensions of cognitive centrality and affective relationship with the reference group (van Zomeren et al., 2008). Being a group identification measure, a reference group is needed; in this case, the *white tide* was the reference group. *White tide* is a group of organizations that defend public health and denounce cuts in Spain.

The measure comprised five Likert scaled items (Cronbach’s alpha = 0.89) that measured feelings of sympathy, identification, connection, and agreement toward the *white tide* (e.g., “I share values and beliefs with the *white tide* movement”).

##### Injustice

Feelings of injustice were measured by asking participants to evaluate the austerity politics performed in the context of public health on three dichotomous scales: unjust–just, bad–good, and negative–positive (Cronbach’s alpha = 0.97). All three scales ranged from 1 to 10.

##### Efficacy

Four items adapted from Hornsey et al. (2006) were used (Cronbach’s alpha = 0.79). Participants were asked to determine the level of efficacy they perceived about protesting in defense of public health in a scale that ranged from *very little effective* to *very effective* (e.g., how effective a protest will be in “*influencing Parliament politicians*”).

##### Affective injustice

Affective injustice was measured by asking people how they felt about the management of the public health system by the government during the crisis [e.g., angry, annoyed, and satisfied (reverse-scored); Cronbach’s alpha = 0.75].

##### Moral conviction

The moral conviction measure for this study was taken from Skitka et al. (2009) and it comprised two items evaluating how nuclear the public health subject was within participant’s moral system (Skitka and Morgan, 2014; e.g., how much are your feelings about this issue connected to your core moral beliefs and convictions?) (Cronbach’s alpha = 0.82).

##### Intention to participate

To measure intention to participate, a shorter version of the instrument used in Study 1 was selected. This time, participants showed their agreement or disagreement on performing five different protest acts (three of them conventional and two unconventional). The scale once again was shown to be reliable (Cronbach’s alpha = 0.84).

### Results

First and foremost, a new CFA of the Moral Obligation Scale was performed; this was done in order to replicate the analysis of the Moral Obligation Scale in Study 1, as a good result will support the robustness of the scale. The model fit for the Moral Obligation Scale was again good: GFI = 0.98; CFI = 0.99; RMSEA = 0.08; and SRMR = 0.01 (see Supplementary Data Sheet S2 for the original data matrix).

A step-wise linear regression analysis was considered to be the best technique to analyze the data. All variables were introduced in the analysis, along with sex and age, in order to control for its influence. The final model [*F*(6,615) = 153.27, *p* < 0.001] showed an *R*^2^ value of 0.59, and resulted in six steps, the variables that significantly predicted intention to participate being (in order from higher to lower β values, criteria that will hold for the rest of the present work): identity, moral obligation, affective injustice, injustice, efficacy, and age (see **Table 3**). Moral conviction was the only variable excluded in the analysis.

**Table 3:** Coefficients of the set of variables used in Study 2 in the prediction of general intention to participate (step-wise linear regression).

	*B*	Std. error	β	*t*	*p*
Identity	0.349	0.040	0.342	8.65	<0.001
Moral obligation	0.273	0.033	0.312	8.23	<0.001
Affective injustice	0.148	0.034	0.134	4.31	<0.001
Injustice	0.083	0.024	0.102	3.48	0.001
Efficacy	0.122	0.047	0.080	2.59	0.010
Age	-0.008	0.003	-0.074	-2.80	0.005

*F*	153.27^∗∗∗^				
*R*^2^	0.59				


*Dependent variable: intention to participate (general). This table represents the coefficients of the last step of the analysis. ^∗∗∗^*p* < 0.001.*

Additionally, as the intention to participate measure comprised both conventional and non-conventional forms of collective action, a step-wise linear regression analysis was performed for both types of participation using the same independent variables.

For conventional participation, the model resulted in five steps [*F*(5,616) = 234.35, *p* < 0.001] with an associated *R*^2^ equal to 0.65. The variables that explained intention in conventional participation were: identity, moral obligation, affective injustice, injustice, and age (see **Table 4**).

**Table 4:** Coefficients of the variables that entered the step-wise analysis predicting for conventional intention to participate.

	*B*	Std. error	β	*t*	*p*
Identity	0.361	0.034	0.377	10.65	<0.001
Moral obligation	0.257	0.028	0.313	9.08	<0.001
Injustice	0.107	0.021	0.140	5.15	<0.001
Affective injustice	0.175	0.030	0.169	5.90	<0.001
Age	-0.006	0.002	-0.060	-2.47	0.014

*F*	234.35^∗∗∗^				
*R*^2^	0.65				


*Dependent variable: intention to participate (conventional). This table represents the coefficients of the last step of the analysis. ^∗∗∗^*p* < 0.001.*

Finally, when non-conventional protest was the dependent variable, the resulting model was configured by four steps [*F*(5,616) = 62.19, *p* < 0.001] that generated an *R*^2^ of 0.33. The independent variables that resulted significantly predictive were as follows: identity, moral obligation, efficacy, affective injustice, and age (see **Table 5**).

**Table 5:** Coefficients of the variables that entered the step-wise analysis predicting for non-conventional intention to participate.

	*B*	Std. error	β	*t*	*p*
Identity	0.370	0.070	0.262	5.29	<0.001
Affective injustice	0.127	0.060	0.083	2.13	0.034
Moral obligation	0.316	0.059	0.261	5.37	<0.001
Efficacy	0.200	0.084	0.095	2.38	0.017
Age	-0.010	0.005	-0.069	-2.05	0.40

*F*	62.19^∗∗∗^				
*R*^2^	0.33				


*Dependent variable: intention to participate (non-conventional). ^∗∗∗^*p* < 0.001.*

A replication of the mediational analysis of Study 1 was performed in order to test if the results held while using a more representative sample. In the case of moral conviction mediated by moral obligation, the ratio of the indirect to total effect was 60% (*P*_M_ = 0.60, SE = 0.07, 95% CI = 0.46, 0.75).

Alternatively, when moral conviction mediated the effect of moral obligation on intention to participate, the effect of the mediation route explained only 10% of the total effect (*P*_M_ = 0.10, SE = 0.02, 95% CI = 0.06, 0.16). As can be observed, results replicated those of Study 1.

### Discussion

The primary aim of this study was to test the performance of the moral obligation variable as a predictor of intention to participate when it was used among other traditional predictive variables of collective action. First, it must be noted that the measure developed in Study 1 for moral obligation proves to be highly reliable again, this time with a more general sample, with Cronbach’s alpha coefficient notably increasing in this second study as well as the CFA indices once more showing a good fit.

The results also support the idea of moral obligation being a robust predictor of the intention to participate in collective action (both conventional and non-conventional), as it showed the second greatest size effect in the first two analyses, topped only by the identity variable and the greatest effect size for non-conventional participation.

Conversely, moral conviction did not feature in any of the three models as a significant predictor of intention to participate in collective action of any kind. This shows the superiority of moral obligation over moral conviction as a predictor of collective action.

Although the principal aim of the current study was not to generate a model for intention of participation, the model that resulted from the analysis came as a remarkably strong one. It accounted for 59% of the variance of the general intention to participate and for 64% of the variance of intention to participate in conventional protest.

As for the rest of variables, in general terms, the present data support previous findings. This can be observed in that all variables related with SIMCA had predictive influence in the general intention to participate. However, efficacy did not significantly predict intention to participate in conventional collective action but did predict intention of non-conventional participation. The reason for this result could be that conventional participation implies fewer potential costs, and thus the perception of efficacy is not so important. On the other hand, non-conventional participation implies greater potential costs, and thus participants will elaborate more in a costs–benefits logic, which will increase the importance of perceived efficacy.

Also, only affective injustice but not the cognitive component of injustice appeared as a predictor of non-conventional intention to participate. This is an unsurprising result as van Zomeren et al. (2008) already showed that affective injustice was a better predictor than cognitive injustice. Additionally, a recent study in collective action in high risk contexts showed that risk perception predicts anger (Ayanian and Tausch, 2016). Although non-conventional actions are not necessarily risky in Western democracies, risk perceptions may be increased, making anger more salient, which could explain that affective injustice overshadows its cognitive component in the prediction of non-conventional collective action intention. Finally, age also had a predictive impact, though it showed the lowest associated effect size of all variables, this effect was also negative, which is congruent with previous findings in the literature (Gallego, 2007).

The present sample has good size and age representation, but it is important to point out that it is overrepresented in terms of women over men, and this constitutes a slight limitation. Another limitation is the use of intention as the dependent variable. There is a well-documented intention–behavior gap, as intentions do not always automatically result in effective behavior (Ajzen et al., 2009). This constitutes another and more important limitation to the present study, making it necessary to test the performance of moral obligation as a predictor variable of real collective action participation, in order to clearly establish its potential explanatory power.

## Study 3

### Introduction

The main goal of the third and last study is to test the predictive power of moral obligation among other variables in the context of a demonstration, comparing people in and outside the protest as well as addressing the limitations of the previous study.

### Method

#### Participants and Procedure

For this study, data were collected *in situ* during the 2017 May Day demonstrations. Thirty-two (32) enquirers worked in the data compilation process. Divided into eight teams of four directed by a pointer. Sampling was carried out following the instructions by Van Aelst and Walgrave (2001) for the gathering of data in a demonstration context. The aim of this procedure is to guarantee that everyone in the demonstration has an equal opportunity to be selected as a participant, eliminating the possible bias of enquirers approaching people that may seem more receptive to them. The procedure consists in forming teams of interviewers, directed by a pointer. Four teams covered the demonstration, starting simultaneously at the top and the back of the demonstration, and worked their way toward the middle, skipping a previously fixed number of rows as they progressed in their respective directions. The pointer decided at all times which demonstrator was to be approached, making the process fully random for the interviewer and allowing for quota control. At the same time, another four teams covered the vicinity of the demonstration in order to get responses of non-demonstrators. This way, the procedure allows to select people that have the possibility to demonstrate but chose to not do it, guaranteeing that they are willingly not demonstrating. Also, the procedure allows for control of certain quotas to increase the chances of getting a highly representative sample. The aim for the sampling process with an expected participation of 9 demonstrators was to obtain a sample of 200 demonstrators, which will be representative for a confidence coefficient of 95% and a sampling error of 7%.

Data were collected using “droidSURVEY” software, as enquirers completed the questionnaire on smartphones, following the commands of participants.

A sample of four hundred and seven (407) participants was gathered, two hundred and sixteen (216) were non-demonstrators, and one hundred and ninety-one (191) were demonstrators. Of the whole sample, two hundred and two participants (202) were women (49.6%). Mean age of the sample was 42.94.

#### Measures

Most variables in Study 3 were the same as in Study 2, and the instruments used to measure them were very similar to those of the previous study. *Moral obligation* was measured using the same scale developed in study 1 and used in study 2. Reliability remained strong (Cronbach’s alpha = 0.84). *Identity* was measured with the same scale as in Study 2, only this time the measure referred to the organizers of the demonstrations instead of referring to the white tide as the reference group. Also, it had four items instead of five (Cronbach’s alpha = 0.92). The *injustice* measure was the same as in Study 2, but in this study, all three scales ranged from 0 to 10 (Cronbach’s alpha = 0.91). The measure for *efficacy* for this study was identical to that of Study 2, where the only difference was an extra item tapping immediate instrumental efficacy of the demonstration (Cronbach’s alpha = 0.83). The a*ffective injustice* measure used the same three items as in Study 2: anger, annoyance, and satisfaction (reversed; Cronbach’s alpha = 0.77).

### Results

As there were some missing values, a multiple imputation was applied to the data to maintain the original sample size. This method was carried out following the recommendations of (Bodner, 2008) (see Supplementary Data Sheet S3 for the original data matrix). A binary logistic regression was conducted with the categorical variable of participant/non-participant in the demonstration as the dependent variable, the rest of measured variables were introduced as independent variables, and age and sex were also introduced as covariables to control its influence. The method chosen for this analysis was the forward Wald method.

Results of the logistic regression were good in terms of significance of the model [χ^2^(5) = 194.05, *p* < 0.001), explained variance (Nagelkerke’s *R*^2^ = 0.50) and classification rate (78.9% of overall correct classification, 77.8% for non-participants, and 80.1% for participants).

The variables that significantly predicted whether people were participants or not participants were as follows: moral obligation, identity, injustice, efficacy, and age. Regarding moral obligation, this value was not only the variable with the highest odds ratio [Exp(*B*) = 2.538], but also almost double the next higher odds ratio, which was that of identity [Exp(*B*) = 1.430; see **Table 6**].

**Table 6:** Variables in the final model of forward Wald binary logistic regression predicting participation vs. non-participation in the May Day demonstration held in Madrid, 2017.

Variable	*B*	Std. error	*Exp*(*B*)	*p*
Moral obligation	0.931	0.151	2.538	<0.001
Identity	0.358	0.095	1.430	<0.001
Injustice	0.268	0.076	1.308	<0.001
Efficacy	0.342	0.146	1.408	0.019
Age	-0.017	0.008	0.983	0.039

χ^2^	194.05^∗∗∗^			
*R*^2^	0.50			


*The table represents the pooled data from the multiple imputation performed to treat missing data. *Exp*(*B*)*: odds ratio*; ^∗∗∗^*p* < 0.001.*

### Discussion

This time, factual participation was measured along a properly comparable group of participants and non-participants in a public demonstration, in an effort to tackle the limitations of Study 2, testing moral obligation in a real environment. The first conclusion we can draw from Study 3 is that moral obligation seems to be a key predictor of participation in demonstrations, as it appears as the predictive variable with the greatest effect size.

This leads us to consider why identity was the most powerful predictor on intention to participate (Study 2) and why moral obligation becomes the most predictive independent variable in the model when factual participation is the dependent variable (Study 3). A possible explanation for this phenomenon could be that moral obligation is more intense when immediate behavior is measured, rather than when the assessment is performed using intention. According to our definition, moral obligation constitutes a motivation to behave according to one’s moral values in order to maintain a positive self-concept. Thus, the more concrete the opportunity to participate becomes, the more the moral obligation will be activated.

As for the SIMCA variables, the only one that did not yield significant impact with participation behavior was affective injustice. Despite affective injustice playing a very central role in mobilizing people (van Zomeren et al., 2008), this may depend on the nature of the demonstration itself. The fact that they are not predictive in this particular case may be because May Day demonstrations have a deep ritual character (Peterson et al., 2012). Ritual actions do not produce that “fire in the belly and iron in the soul” that Gamson (1992, p.32) describes when he refers to anger. As for sociodemographic variables, age again was a significant predictor with a negative relation on participation. Nevertheless, its influence is negligible compared to the rest of the variables used in the analysis.

## General Discussion

The main goal of this paper was to conceptualize and test the predictive power of moral obligation in the context of collective action participation. In order to do this, a scale for the measure of moral obligation was developed, which is believed to represent a better balance between length and exhaustiveness than previous ones. Another objective was to check if moral obligation is any different to other concepts such as moral norm and moral conviction, which have a strong presence in the literature. Our results confirm that they are three independent concepts. Nevertheless, it is more important to stress the fact that moral norm and moral conviction play their part as antecedents of moral obligation. This disentangles the conceptual disarray which ensues when discussing these three moral concepts. The data presented here not only discourage us from treating them as synonyms, but also seem to indicate that in the attitude–behavior continuum; the moral scheme is as follows: norm–conviction–obligation.

However, without a doubt, the central topic of the present research was to test the predictive capacity of moral *obligation* over collective action participation. In order to do so, this variable underwent a double “benchmarking process.” First, its effects on collective action participation were measured along with another moral variable already proven to be efficient in the prediction of this behavior, moral conviction (van Zomeren et al., 2012). Results clearly point toward moral obligation as having greater predictive capacity on collective action participation. Equally relevant is the fact of having shown – without forgetting to exercise caution, as is customary in correlational studies – that moral conviction precedes moral obligation.

The second benchmark that moral obligation was put through was the testing of its predictive effects when featuring along with the SIMCA (identity, injustice, and efficacy) variables in the statistical analyses. Results were again encouraging for moral obligation. In Study 2, where the intention to participate was analyzed, it played a key role, almost to the level of identity. In Study 3, where a real demonstration was the object of study, it was the variable that produced the greater effect size, followed distantly by identity. It is important to note that identity is, in all likelihood, the most relevant variable in collective action studies. The role that is assigned to it in the SIMCA model is a good testament to that (van Zomeren et al., 2008). The fact that moral obligation showed a very similar predictive capacity – or even sometimes greater – prompts us to consider introducing moral along with the classical paths of identity, injustice, and efficacy as the main motives for collective action participation. This means bringing the moral dimension of human behavior back to the spotlight, which for some time was relegated to a minor role due to the primacy of consequentialist approaches.

Another aspect that must not be forgotten is that the influence of moral obligation over participative behavior in collective action provides useful practical insight beyond the more scientific interest it may have. It gives useful information to the organizers of demonstrations, as promoting moral obligation could help them attract more people to these acts. Moreover, it seems to be a useful way to promote other kinds of behavior related to moral considerations. For instance, framing a specific issue (e.g., climate change) as a moral one and the behavior to promote an issue (e.g., recycling) as a moral obligation could allure people to engage in that given action. There have already been enticing proposals on how to promote specific kinds of collective actions (Guizzo et al., 2017), and we believe that the promotion of a sense of moral obligation could substantially aid promotional efforts.

## Limitations of the Present Research and Future Directions

As the present research has been performed entirely with Spanish samples, the generalization of the findings to other contexts remains to be tested. Political participation in general and protest participation in particular are susceptible to contextual differences (Klandermans et al., 2002). From here, we encourage future research to replicate our findings abroad, to determine if moral obligation is indeed a relevant, transcultural predictor of collective action. The cultural context where protest takes place and/or its subject could as well generate some differences (Ketelaars et al., 2014; Sabucedo et al., 2017b). Additionally, both scenarios of participation considered in this research are limited to pacific actions (even when non-conventional intention is measured) and also to actions that look to solve in-group grievances. The fact that moral obligation implies a motivational pressure to defend one’s moral beliefs, even when facing personal costs, posits moral obligation as a potential useful predictor of other kinds of actions like humanitarian (Thomas et al., 2016) or even violent ones (Saab et al., 2016).

Another interesting puzzle for future research could be to determine under what circumstances moral obligation may be overridden. Although moral obligation seems to be a very powerful motivation, there are times when people will go against it even though that will, in theory, translate into a devaluated self-concept. This devaluation is not likely to happen however, as people will resort to cognitive strategies in order to reduce cognitive dissonance and self-discrepancy, such as looking for external causes for non-compliance (Greenwald and Banaji, 1995). The exploration of these compensatory strategies could have great practical implications, as it allows organizers to know what thoughts people use to overpass moral obligation, and thus argue against them. Additionally, what is probably the most exciting perspective for future research on the collective action field is the integration of moral obligation into a predictive model of collective action.

Now, to bring this paper to a close, it has to be said that we believe the present research establishes moral and particularly moral obligation as one of the main motivations for participating in collective action. Moral obligation guarantees that convictions and principles are not just mere cognitive-moral referents that expresses themselves in favorable conditions, but are inhibited in contexts that may imply high personal costs. In crisis situations, as denounced by Dante Alighieri, confirmed by Hanna Arendt, and outstanding social psychologists have shown, it is not uncommon to find citizens who opt for indifference, “neutrality” or looking the other way. Staub (2013) is explicit in pointing out that passivity and complicity are often the same thing. In this sense, moral obligation allows for engaging in collective actions aimed at combating, for example, injustice and social inequality that threatens, as the World Health Organization reminds us, the welfare of citizens and communities. Thus, we avoid going to the worst place in hell, reserved, as the Florentine poet promised, for those who do not involve themselves with the problems of their time.

## Ethics Statement

According to The APA Ethic Code, participants were informed about: (a) the purpose of research, its estimated duration, and procedures; (b) their right to decline participation or withdraw it at all times; (c) the minimum potential risks this study implied; (d) the confidentiality of the data, that was guaranteed as the software used to gather the data allowed the researchers to make responses non-trackable, as it was indeed done; (e) whom to contact for questions about the research and research participants’ rights. Considering this, the present study meets the Ethical principles of psychologists and code of conduct proposed by the American Psychological Association (2002) as well as those ethical regulations made by the Ethical Committee of the University of Santiago de Compostela for Social Science studies with people, that is, fulfilling the requirements of informed consent and data protection (Organic Law 15/1999). Also, this work included a deception in Study 1. The deception was considered necessary in order to place participants in a “pro-protest” setting. Participants were informed of this deception as soon as their participation had ended.

## Author Contributions

All the referred authors made significant contribution to the present study. J-MS and MD took part in the conception, design, and data analysis. MD also contributed with the drafting of the paper and the data gathering. J-MS and MA worked in the critical review of the article and wrote sections of it. GS contributed to performing specialized data analysis. All authors contributed to manuscript revision, read, and approved the submitted version.

## Conflict of Interest Statement

The authors declare that the research was conducted in the absence of any commercial or financial relationships that could be construed as a potential conflict of interest.
