# Supplementary material for: The Importance of Protesters’ Morals: Moral Obligation as a Key Variable to Understand Collective Action
Source: Front Psychol. 2018 Mar 27;9:418. doi: 10.3389/fpsyg.2018.00418 (PMC5881521; doi:10.3389/fpsyg.2018.00418)
Supplement: Supplementary file 4 [file Data_Sheet_4.docx]

**Appendix I. Moral Scales.**

**Moral obligation**

1. To mobilise against [subject of mobilization] constitutes a moral obligation to oneself.
   *Movilizarse contra […] constituye una obligación moral para con uno mismo.*
2. To mobilise against […] would make me feel proud of myself.
   *Movilizarme […] me haría sentir orgulloso/a de mí mismo/a.*
3. To not mobilise against […] would make me feel guilty.
   *No movilizarme […] me haría sentir culpable.*
4. I feel morally obliged to mobilise against […] even when that means confronting people that are close to me.
   *Me siento moralmente obligado/a a movilizarme […] incluso si ello supone enfrentarme a personas cercanas a mí.*
5. No matter what anyone thinks, I feel morally obliged to participate in demonstrations […].
   *Independientemente de lo que los demás piensen, me siento moralmente obligado/a a participar en movilizaciones […].*

**Moral norm**

1. I consider morally correct to participate in mobilizations for/against […].
2. To organise with others to fight for/against […] is something morally correct.
3. I consider morally correct to initiate personal actions to fight for/against […].

**Moral conviction**

1. My opinion about […] is important to me.
2. I believe that my opinion about […] is an important part of my moral norms and values.
3. I believe that my opinion about […] has a moral character.
4. My opinion about […] reflects an important part of who I am.
